# Health Information Seeking From an Intelligent Web-Based Symptom Checker: Cross-sectional Questionnaire Study

**DOI:** 10.2196/36322

**Published:** 2022-08-19

**Authors:** Kimberly Arellano Carmona, Deepti Chittamuru, Richard L Kravitz, Steven Ramondt, A Susana Ramírez

**Affiliations:** 1 School of Social Sciences, Humanities and Arts University of California Merced, CA United States; 2 University of California, Davis Sacramento, CA United States; 3 Department of Donor Medicine Research Sanquin Research Amsterdam Netherlands; 4 Department of Communication Science Vrije Universiteit Amsterdam Amsterdam Netherlands

**Keywords:** health information seeking, health information, information seeking, information seeker, information behavior, artificial intelligence, medical information system, digital divide, information inequality, digital epidemiology, symptom checker, digital health, eHealth, online health information, user demographic, health information resource, health information tool, digital health assistant

## Abstract

**Background:**

The ever-growing amount of health information available on the web is increasing the demand for tools providing personalized and actionable health information. Such tools include symptom checkers that provide users with a potential diagnosis after responding to a set of probes about their symptoms. Although the potential for their utility is great, little is known about such tools’ actual use and effects.

**Objective:**

We aimed to understand who uses a web-based artificial intelligence–powered symptom checker and its purposes, how they evaluate the experience of the web-based interview and quality of the information, what they intend to do with the recommendation, and predictors of future use.

**Methods:**

Cross-sectional survey of web-based health information seekers following the completion of a symptom checker visit (N=2437). Measures of comprehensibility, confidence, usefulness, health-related anxiety, empowerment, and intention to use in the future were assessed. ANOVAs and the Wilcoxon rank sum test examined mean outcome differences in racial, ethnic, and sex groups. The relationship between perceptions of the symptom checker and intention to follow recommended actions was assessed using multilevel logistic regression.

**Results:**

Buoy users were well-educated (1384/1704, 81.22% college or higher), primarily White (1227/1693, 72.47%), and female (2069/2437, 84.89%). Most had insurance (1449/1630, 88.89%), a regular health care provider (1307/1709, 76.48%), and reported good health (1000/1703, 58.72%). Three types of symptoms—pain (855/2437, 35.08%), gynecological issues (293/2437, 12.02%), and masses or lumps (204/2437, 8.37%)—accounted for almost half (1352/2437, 55.48%) of site visits. Buoy’s top three primary recommendations split across less-serious triage categories: primary care physician in 2 weeks (754/2141, 35.22%), self-treatment (452/2141, 21.11%), and primary care in 1 to 2 days (373/2141, 17.42%). Common diagnoses were musculoskeletal (303/2437, 12.43%), gynecological (304/2437, 12.47%) and skin conditions (297/2437, 12.19%), and infectious diseases (300/2437, 12.31%). Users generally reported high confidence in Buoy, found it useful and easy to understand, and said that Buoy made them feel less anxious and more empowered to seek medical help. Users for whom Buoy recommended “Waiting/Watching” or “Self-Treatment” had strongest intentions to comply, whereas those advised to seek primary care had weaker intentions. Compared with White users, Latino and Black users had significantly more confidence in Buoy (*P*<.05), and the former also found it significantly more useful (*P*<.05). Latino (odds ratio 1.96, 95% CI 1.22-3.25) and Black (odds ratio 2.37, 95% CI 1.57-3.66) users also had stronger intentions to discuss recommendations with a provider than White users.

**Conclusions:**

Results demonstrate the potential utility of a web-based health information tool to empower people to seek care and reduce health-related anxiety. However, despite encouraging results suggesting the tool may fulfill unmet health information needs among women and Black and Latino adults, analyses of the user base illustrate persistent second-level digital divide effects.

## Introduction

### Background

The ever-growing amount of health information available on the web is increasing the demand for tools that provide personalized and actionable health information. In addition, patients avidly seek information to inform their own health care decisions, either directly or by verifying information discussed during professional consultations. The broad scope of web-based health information includes generic information obtained through web-based searches and decision aids and tools that deliver personalized advice based on information specific to users. Such tools include symptom checkers that provide users with a potential diagnosis after responding to a set of probes about their symptoms.

Web-based symptom checkers are becoming increasingly popular, and the emergence of the COVID-19 pandemic has increased interest in these tools [1]. However, only a few studies have examined how and why they are used [2-4]. The limited research on symptom checkers has found generally positive effects of their use; technologically sophisticated web-based triage systems may help reduce unnecessary visits to emergency rooms and overuse of antibiotics [4], make health care accessible in low-resource settings [5], and increase patient engagement [6]. However, although the potential for their utility is great, more research is needed on the actual use and effects of such tools.

Some studies have raised concerns about the potential of web-based health information systems to spread disinformation and inaccurate diagnostic information [2,7,8]. For example, a study evaluating the diagnostic and triage accuracy of 23 web-based symptom checkers found that physicians performed better than the symptom checker algorithms [4]. However, physicians made incorrect diagnoses in 15% of the cases. Although research suggests that symptom checkers may be less effective than physicians in terms of diagnostic accuracy, it might be more critical that symptom checkers provide recommended actions (eg, whether symptoms warrant a trip to the hospital). Therefore, it is important to understand the impact of symptom checkers on how patients seek care and respond to health care advice.

A significant potential contribution of web-based symptom checkers as triage systems may be to reduce the negative effects of the current overwhelming health information environment, such as the health information overload experienced by web-based health information seekers and their struggle to discern reliable information from misinformation. A web-based medical information system that addresses the abovementioned problems can help people better understand the potential causes of the symptoms they are experiencing, empower them to seek the right kind of help, and potentially reduce anxiety caused by the symptoms they are experiencing.

Users must be able to trust and follow their recommendations for web-based symptom checkers to make meaningful contributions. If web-based symptom checkers are not trusted, they are less likely to be adopted by users, thereby limiting their potential [9]. Moreover, users may be unclear about the technology behind web-based symptom checkers. Research suggests that web-based symptom checkers’ artificial intelligence (AI) systems are neither transparent nor comprehensible to users, which may undermine trust in such tools [10]. Nevertheless, despite hesitancy and concerns regarding the accuracy, AI-powered symptom checkers have been perceived as useful for diagnosis by users [11].

A large body of research on information seeking grounded in the uses and gratification frameworks [12] has examined how people use different media to fulfill or gratify various needs. Research in this tradition has characterized health information–seeking behaviors by sources (ie, web-based vs offline seeking [13,14]) or objective (ie, seeking for themselves vs others [15-17]). Multiple studies have confirmed that active information seekers from nonclinical sources, including the internet, are more likely to be White, female, and have relatively high levels of education and income [18-22]. Racial differences in health information–seeking, as well as confidence in information and trust in various sources, have been well documented. There may be different levels of trust and use of sources by racial groups, which can lead to disparities if inaccurate sources are used [23].

Research based on self-reported media use has established that deliberate information seeking from media, including the internet, has been associated with better health outcomes [24], increased engagement in prevention behaviors [25], and more positive patient-clinician interactions [26-28] and has also assisted individuals in coping with uncertainty [20]. Web-based health information–seeking before presenting to an emergency physician also has the potential to improve patient-provider interaction without negatively affecting adherence to treatment [29].

Despite this extensive body of research on information seeking and the importance of the internet and other “new” media as sources of medical information, the quality of the evidence for the effects has been limited. Most previous studies examining information seeking from nonclinical sources, including nearly all internet-based health information–seeking studies, are limited by their reliance on self-reports of individuals’ information-seeking behaviors and behavioral or psychosocial outcomes. Furthermore, most studies rely on generalized, non–time-bound health information–seeking behaviors (ie, “Have you ever looked for information about [a topic] from [a source]”), or ask about information seeking within a specific timeframe, but do not examine the content of the information retrieved or the recommendation provided. Thus, the next frontier in this line of research is one that links objective measures of information seeking—both sources and content—with clinical and psychosocial outcomes to understand how people use the information they seek and find from nontraditional sources.

### Objectives

This study aimed to address the methodological limitations of prior information-seeking research and examine who seeks information from an intelligent web-based symptom checker and for what purpose, how users experience the tool, what they intend to do with the information, and predictors of intentions to follow the tool recommendations. The following research questions (RQs) guided this study:

1. RQ1: Who uses a web-based symptom checker?
2. RQ2: What drives users to use a web-based symptom checker?
3. RQ3: What were the web-based symptom checker’s recommendations?
4. RQ4: How do users perceive the web-based symptom checker?
5. RQ5: What is the relationship between perceptions of a web-based symptom checker and intention to follow recommended actions?

## Methods

We conducted a cross-sectional survey of web-based health information seekers immediately following the completion of a visit to a web-based intelligent symptom checker, Buoy Health (Buoy Health, Inc [30]; N=2437).

### Buoy Health: an AI-Powered Web-Based Symptom Checker

**Figure 1:**
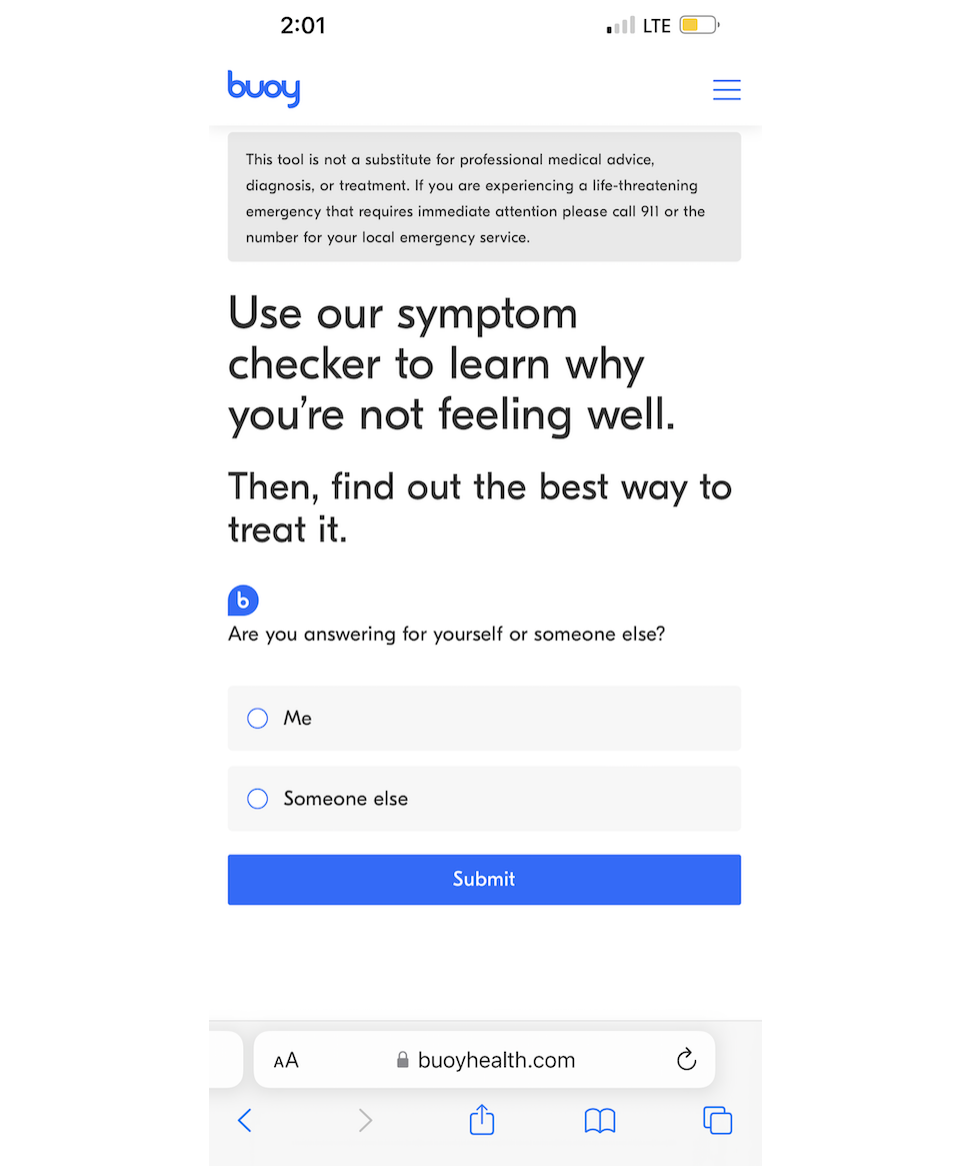
Screenshot of the patient-facing, artificial intelligence–assisted Buoy Health symptom checker.

This cross-sectional study used data from patient encounters using Buoy Health, an AI-powered web-based symptom checker, between January 14, 2019, and February 28, 2019. Founded in 2014 by a team of physicians and researchers, the tool is based on conversational medical interviewing, mirroring a conversation with a provider. At the time of writing, Buoy’s symptom checker remains accessible for free on the web or through an app to any internet-connected person. The AI-powered tool uses a progressive series of health questions communicated via a chatbot to assess user symptoms (Figure 1). Buoy’s triage or diagnostic system by design offers health information customized for the user.

**Figure 2:**
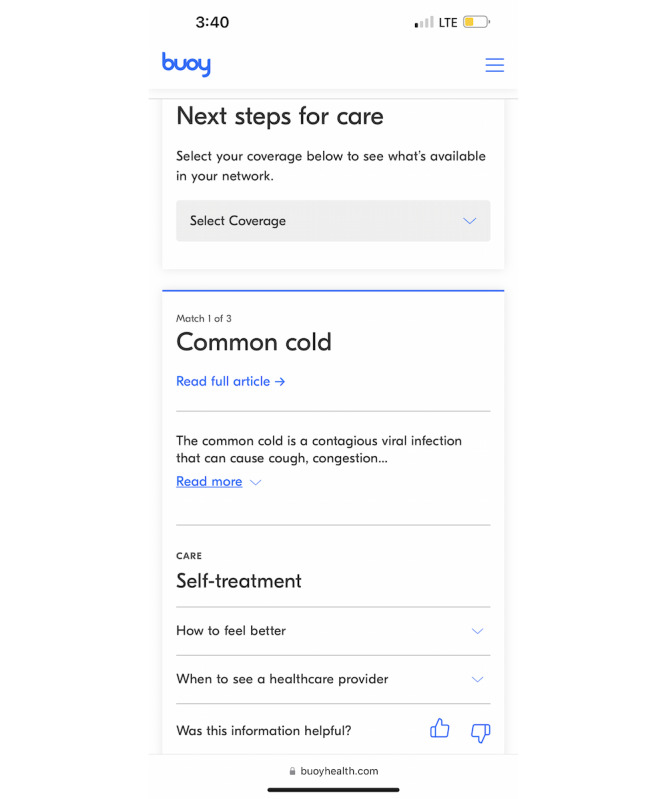
Screenshot of a Buoy Health symptom checker recommendation.

Buoy’s proprietary algorithm sources data from >18,000 clinical research studies [31]. Users receive 3 possible diagnoses and recommendations for appropriate levels of care (Figure 2). According to Buoy, the tool’s diagnostic accuracy is 90% [32]. Thus, tools such as Buoy—and other intelligent symptom checkers—have the potential to cut through the clutter of too much and contradictory information to provide personalized, science-based recommendations. A study examining how patients’ use of Buoy affected their plans for seeking care found that Buoy decreased uncertainty among users [33]. Buoy also lowered the level of urgency in patients associated with their condition. This study suggests tools such as Buoy are associated with users’ intended behavior when seeking care based on triage questions. Accordingly, our study adds to the growing literature that seeks to understand how patients use tools such as Buoy together with their providers to manage their health.

### Sampling and Procedure

A recruitment script was shown to Buoy users, assumed to be web-based health information seekers, who met the inclusion criteria via a pop-up window immediately following their Buoy session. Inclusion criteria included completion of the Buoy interview to the recommendation stage in ≤10 minutes, being aged ≥18 years, and residency in the United States (although not necessarily physically in the United States at the time of seeking).

**Figure 3:**
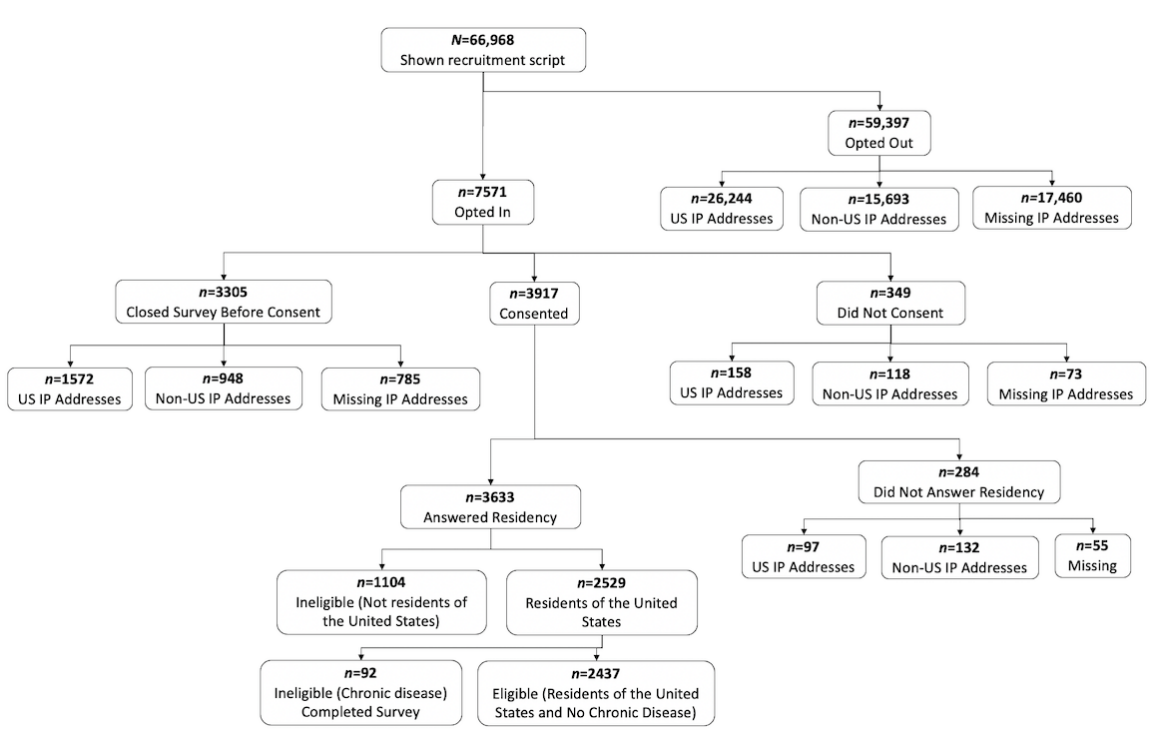
Flow diagram showing attrition of participants.

In addition, potential participants must have completed the Buoy interview for themselves; that is, they were seeking information about their symptoms. As 95% of Buoy users complete the diagnostic interview within 10 minutes, users who took longer were not representative of the typical user and thus were not invited to participate in our study to avoid other unanticipated ways in which they might differ from the typical user. Using similar logic, we excluded people who had a pre-existing serious or chronic condition [34] as they may not be representative of the typical Buoy user either. It is expected that their health information–seeking habits and use patterns of Buoy would be different from all other Buoy users. Finally, for ethical reasons, we excluded users who Buoy advised to seek immediate medical care from eligibility, including immediate medical care via 911 or in the emergency department. Figure 3 shows attrition at each stage.

Participants received a US $5 electronic gift card in appreciation of their time following completion of the survey, which had a mean time to completion of 8.61 (SD 6.78) minutes. The gift cards were delivered to an email address that was also used for follow-up. Participants were informed that they would receive another incentive (US $10) following the completion of a second survey. A follow-up assessment was administered 2 weeks after the initial survey to those who chose to provide an email address; however, this study reports only the baseline data.

### Constructs and Measures

#### Overview

This study followed the tradition of uses and gratifications research [12]. We sought to understand who uses Buoy, perceptions of the user experience, and what they intend to do with the information they obtain. The survey was guided by the integrative model of behavior change [35]. The key constructs and measures are described in the following sections, and the complete survey instrument is available upon request from the corresponding author.

#### Reasons for Using Buoy

Patients could select ≥1 of 5 reasons for using Buoy; the list was based on the review of internet use for appraisal of symptoms of physical illness by Mueller et al [3]. Options included not being confident that the health care provider provided the correct diagnosis, symptoms not serious, sensitive or embarrassing symptoms, new symptoms, and persistent symptoms. An open-ended response was also provided, and the results were interpreted by 2 coders to map to original close-ended or new codes (access, anxiety, curiosity, and triage).

#### Trust in Health Information Sources

Trust in a variety of health information sources was assessed using a single Likert item, with responses ranging from “Not at all” (1) to “A lot” (4), adapted from the National Cancer Institute’s Health Information National Trends Survey [36]. The question stem was “In general, how much would you trust information about health or medical topics from each of the following?” The list of sources was randomized: physician/family or friends/newspapers or magazines/radio/internet news/television/government health agencies/social media (such as Facebook or Twitter)/Google/blogs/Buoy.

Prior research has demonstrated that the usability of the health information website affects trust in and credibility of the health information found on the site [37]. Thus, we assessed confidence, comprehensibility, perceived utility, and the emotional effects of using Buoy.

#### Confidence in Smart Symptom Checkers

A total of 2 items with 5-point response values from “Not at all confident” to “Very Confident” were adapted from Sivakumar et al [38] and combined as a scale where higher values represented greater confidence.

#### Comprehensibility of Smart Symptom Checkers

We assessed the extent to which the language on the website was easy to understand and the website was understandable and easy to read using 3 response values of 5 points (strongly disagree to strongly agree). Items were based on a scale by van Weert et al [39], with higher values representing greater comprehensibility of Buoy.

#### Perceived Utility of Smart Symptom Checkers

A total of 3 items with 5-point response values (strongly disagree to strongly agree) and combined as a scale by Davis [40] were used to assess the extent to which the website made the diagnosis of symptoms quicker and easier and the website’s overall usefulness.

#### Emotional Effects of Using Smart Symptom Checkers

The emotional effect was informed by White and Horvitz [41] and was measured using a scale of 2 items of 5 points (strongly disagree to strongly agree). The items assessed anxiousness about a perceived medical condition and the extent to which the website encouraged help seeking. Higher values represented more positive emotional effects of using the website.

#### Recommended Action

At the conclusion of the Buoy diagnostic interview, users were given at least one and up to 3 of 8 possible recommendations for the next steps (triage level) that correspond to their cluster of symptoms and potential diagnosis: (1) wait and watch, (2) self-treatment, (3) phone call or in-person visit in the next 3 days, (4) primary care physician in 2 weeks, (5) primary care physician in 1 to 2 days, (6) in-person visit that day or as soon as possible, (7) hospital emergency room, and (8) emergency medical service. Buoy users who received the 2 most urgent recommended actions were not included in our study for ethical reasons. A complete description of the recommendations is available in Multimedia Appendix 1. Buoy provided the research team with the actual recommendations shown to all eligible users. In addition, we asked participants to indicate which of the 6 possible recommendations they had received from Buoy. We compared participants’ self-reports with the Buoy-reported recommendations as a manipulation check. The comparison matched survey responses with at least one of the Buoy recommendations. Most self-reported recommendations matched at least one recommendation, as reported by Buoy (1595/2141, 74.49%).

#### Intention to Follow Recommended Action

The reasoned action approach informed this measure [35]. Intention was measured for all 6 included recommended actions and intentions to discuss Buoy’s recommendation with a physician or other health professional. The response values ranged from 1 (strongly disagree) to 5 (strongly agree). Examples of the statement are as follows, “I intend to [follow Buoy’s recommendation]” and “I intend to discuss the information I got from Buoy with my doctor or other health care professional.” Higher values on the item represented a stronger intention to follow Buoy’s recommendations or discuss the same with the physician. The recommended action was assessed as a binary variable. Users who scored 4 or 5 on intention (high) were classified as having medical intention, whereas those who scored 1, 2, or 3 were classified as having no medical intention. Intention to follow “Wait and Watch” and “Self-Treatment” were combined on a “No Medical Intention” scale. The intentions “Phone Call or In-Person Visit in the next 3 Days,” “Primary Care Doctor in 1-2 days,” and “In-Person Visit Today or ASAP” were combined in a “Medical Intention” scale.

### Coding of Symptoms and Diagnoses

Users’ self-reported symptoms resulting from the Buoy interview were coded into 13 categories using the Centers for Disease Control and Prevention National Ambulatory Medical Care Survey (NAMCS) coding protocol [42]. During the interview, the users were prompted to enter up to 5 presenting symptoms. We report only on the first as that was the primary issue driving the use of the web-based symptom checker. Using an iterative coding process, we generated a set of unique symptoms (N=2040) and unique diagnoses (N=938) from all Buoy data sets.

A total of 2 coders independently coded the first symptom. Coder 1 was part of the codebook development process. Coder 2 was introduced into the study once the codebook was finalized. Disagreements were resolved by discussion. The second author resolved disagreements when consensus was not possible. Cohen κ was run to determine interrater reliability between the 2 coders’ assignment of NAMCS codes for the 2040 unique symptoms; there was substantial agreement between the 2 coders (κ=0.73; [43]). We further categorized whether the first symptoms were serious and likely to require medical attention based on Shapiro et al [44] (chest pain that is heart related, bleeding, loss of consciousness, shortness of breath, and weight loss).

Users are provided with up to 3 possible diagnoses or display names at the completion of their interview, ranked and weighted according to Buoy’s proprietary algorithm, along with recommendations for subsequent actions. Diagnoses were coded into 25 categories comprising major systems, disorders, and conditions, in line with the NAMCS. We report the first diagnosis display name as the algorithm had the highest confidence in it. In addition, the first diagnosis display name had no missing data.

### Analytic Approach

For this descriptive analysis, we computed frequencies and percentages to summarize participant characteristics and experiences, overall and by sex and ethnicity where relevant, and to assess intentions to comply with Buoy recommendations. ANOVAs with Bonferroni correction examined the mean outcome differences between racial or ethnic groups on user experience and recommendations. Nonparametric tests in the form of the Wilcoxon rank sum test were performed to test the mean differences between sexes in user experience and recommendations. The relationship between perceptions of the symptom checker and intention to follow recommended actions was assessed using logistic regression. Logistic regression models examined the factors affecting confidence in recommendations and intention to follow these. Analyses were conducted using R (version 4.0.3; R Foundation for Statistical Computing).

### Ethics Approval

The University of California, Merced Institutional Review Board approved this study (approval number: UCM2018-124).

## Results

### Users of a Web-Based Symptom Checker

Consistent with prior studies on health information seekers, Buoy users were well-educated (1384/1704, 81.22% some college or more), mostly White (1227/1693, 72.47%), and female (2069/2437, 84.89%). The mean age of the users was 39.4 (SD 14.7) years. Users were similar to other users of web-based symptom tools, and a prior study of web-based symptom checkers found that users were predominantly female with a mean age of 40 years [33]. Findings from other studies further indicate an age, sex, and socioeconomic divide among adults’ web-based health information–seeking behaviors [45]. The sampled users were also relatively privileged in terms of health care access; most had insurance (1449/1630, 89%) and a regular health care provider (1307/1709, 76%). They were generally in good health; 59% (1000/1703) reported their health as good, very good, or excellent. Table 1 shows the additional demographic details.

**Table 1:** Sample characteristics and comparison with all users of an intelligent web-based symptom checker.

Characteristics		Analytic sample^a^ (N=2437)	Eligible opt-outs (N=27,816)
**Age (years)**			
	Values, mean (SD)	39.35 (14.43)	36.92 (14.13)
	Values, range	18-87	18-89
**Ethnicity (N=1693), n (%)**			
	White	1227 (72.47)	—^b^
	Black or African American	189 (11.16)	—
	Latino or Hispanic	139 (8.21)	—
	Asian or Pacific Islander	86 (5.08)	—
	Other	52 (3.07)	—
**Education (N=1704), highest level completed, n (%)**			
	High school or less	320 (18.78)	—
	Some college	689 (40.43)	—
	College	695 (40.79)	—
**Household income (US $; N=1654), n (%)**			
	<20,000	304 (18.38)	
	20,000-34,999	226 (13.66)	—
	35,000-49,999	232 (14.03)	—
	50,000-74,999	316 (19.11)	—
	75,000-99,999	237 (14.33)	—
	≥100,000	339 (20.50)	—
**General health status (self-reported; N=1703), n (%)**			
	Excellent	63 (3.70)	—
	Very good	288 (16.91)	—
	Good	649 (38.11)	—
	Fair	532 (31.24)	—
	Poor	171 (10.04)	—
Have regular health care provider (N=1709), n (%)		1307 (76.48)	—
Have insurance (N=1630), n (%)		1449 (88.90)	—

^a^The number of Buoy users in the analytic sample was 2437; during the period of the study, there were a total of 27,816 potentially eligible users (aged ≥18 years, US IP address, those seeking for themselves, and who completed the Buoy interview in <10 minutes) who opted not to participate.

^b^Data not available.

### Drivers for Users to Use a Web-Based Symptom Checker

Users selected ≥1 of the 5 stated reasons for using Buoy, as well as open-ended responses, which were coded into 5 new categories. Over one-third (839/2437, 34.43%) of the users indicated persistent symptoms as a reason for using Buoy, followed by new symptoms (767/2437, 31.47%), symptoms not serious (545/2437, 22.36%), sensitive or embarrassing symptoms (269/2437, 11.04%), and not confident that health care provider provided correct diagnosis (220/2437, 9.03%). Less common reasons included new categories/codes: curiosity (66/2437, 2.71%), access (36/2437, 1.48%), anxiety (16/2437, 0.66%), triage (29/2437, 1.19%), and other (30/2437, 1.23%; data not shown).

### Recommendations of the Web-Based Symptom Checker

We report the patterns in symptoms and diagnoses in 2 ways. First, we report the frequencies of symptoms and diagnoses organized by the NAMCS Biological Systems associated with them (Multimedia Appendix 2 [42,44]). Second, we report the top 10 symptoms and diagnosis categories, overall and by sex and race/ethnicity (Table 2).

**Table 2:** Top 10 symptoms and diagnoses (sorted into major categories), overall and by sex and ethnicity (N=2437).

Symptoms and diagnoses				Overall		Female (n=2069)		Male (n=368)		White (n=1227)		Latino (n=139)		Black (n=189)		Asian or Pacific Islander (n=86)
**Primary symptom, proportion**																
	**Musculoskeletal pain; headache; other pain**		0.35		0.34		0.42		0.40		0.31		0.21		0.21	
		Axial musculoskeletal pain	0.05		0.05		0.06		0.06		0.02		0.02		0.01	
		Muscle pain	0.06		0.06		0.08		0.07		0.06		0.03		0.03	
		Joint pain	0.08		0.08		0.09		0.09		0.07		0.03		0.07	
		Headache	0.03		0.03		0.02		0.03		0.02		0.02		0	
		Chest pain	0.02		0.02		0.03		0.02		0.03		0.02		0.01	
		Other pain	0.11		0.10		0.13		0.11		0.10		0.08		0.08	
	Gynecological problems		0.12		0.14		0.00		0.08		0.16		0.22		0.26	
	All masses, lumps, and tumors		0.08		0.07		0.13		0.09		0.07		0.10		0.09	
	Edema		0.05		0.05		0.05		0.05		0.06		0.05		0.03	
	Skin issues		0.05		0.04		0.09		0.05		0.06		0.08		0.08	
	Gastrointestinal problems		0.05		0.05		0.04		0.06		0.10		0.03		0.06	
	Impaired sensation		0.04		0.03		0.06		0.04		0.01		0.04		0.03	
	Urinary tract problems		0.03		0.03		0.02		0.03		0.03		0.04		0.03	
	Acute upper respiratory tract symptoms		0.03		0.04		0.01		0.03		0.05		0.02		0.03	
	Other		0.18		0.19		0.17		0.19		0.15		0.22		0.16	
**Primary diagnosis, proportion**																
	Musculoskeletal conditions		0.12		0.12		0.17		0.14		0.08		0.07		0.07	
	Musculoskeletal injuries		0.05		0.05		0.05		0.06		0.04		0.04		0.02	
	Gynecological conditions		0.12		0.15		0		0.09		0.17		0.22		0.20	
	Skin problems		0.12		0.11		0.17		0.12		0.09		0.15		0.14	
	Infectious diseases		0.12		0.13		0.10		0.13		0.19		0.09		0.13	
	Digestive conditions		0.07		0.07		0.08		0.08		0.07		0.05		0.06	
	Neurological conditions		0.07		0.07		0.08		0.09		0.08		0.05		0.10	
	Cancer and benign growths		0.05		0.04		0.05		0.04		0		0.02		0.01	
	Urination problems		0.03		0.04		0.02		0.03		0.04		0.04		0.05	
	Endocrinal problems and conditions		0.03		0.03		0.02		0.03		0.01		0.04		0.03	
	Heart related issues		0.02		0.02		0.03		0.02		0.01		0.03		0.01	
	Other		0.17		0.18		0.22		0.18		0.24		0.19		0.17	

Only 3 types of symptoms—pain (855/2437, 35.08%), gynecological issues (293/2437, 12.02%), and masses or lumps (204/2437, 8.37%)—accounted for almost half (1352/2437, 55.48%) of the site visits. The top 3 symptoms entered by men included pain (154/368, 41.8%), masses or lumps (49/368, 13.3%), and skin issues (33/368, 8.9%), whereas the top 3 symptoms in women included pain (701/2069, 33.88%), gynecological issues (293/2069, 14.16%), and masses or lumps (155/2069, 7.49%). Pain, gynecological issues, and masses or lumps were also reported as the top 3 symptoms for White, Black, and Asian or Pacific Islander users. The top 3 symptoms in Latino users were pain (43/139, 30.9%), gynecological issues (22/139, 15.8%), and gastrointestinal problems (14/139, 10.1%). In comparison, Native Americans, who represented <1% of users, only entered five symptoms: pain (4/13, 31%), gynecological issues (4/13, 31%), skin issues (1/13, 8%), gastrointestinal problems (1/13, 8%), and impaired sensation (1/13, 8%).

Among the entire sample, major diagnoses were musculoskeletal (303/2437, 12.43%), gynecological (304/2437, 12.47%) and skin conditions (297/2437, 12.19%), and infectious diseases (300/2437, 12.31%). Comparably, the top 3 diagnoses reported by Buoy for men included musculoskeletal conditions (63/368, 17.1%) and skin conditions (62/368, 16.8%) and infectious diseases (37/368, 10%). The top 3 diagnoses for women included gynecological conditions (304/2069, 14.69%), infectious diseases (263/2069, 12.7%), and musculoskeletal conditions (240/2069, 11.59%). The diagnoses based on race or ethnicity followed a similar pattern. White users also reported musculoskeletal conditions (177/1227, 14.42%), infectious diseases (163/1227, 13.28%), and skin conditions (148/1227, 12.06%) as the top 3 diagnoses. Latino, Black, and Asian or Pacific Islander users reported gynecological conditions, skin conditions, and infectious diseases as the top 3 diagnoses. Gynecological conditions were reported as the top diagnosis category by Black (42/189, 22.2%) and Asian or Pacific Islander (17/86, 20%) users, whereas Latino users reported infectious diseases (26/139, 18.7%) as the top diagnosis category.

Buoy’s primary recommendation was more evenly split across the less-serious triage categories. Users self-reported primary care physicians in 2 weeks (754/2141, 35.22%), self-treatment (452/2141, 21.11%), and primary care in 1 to 2 days (373/2141, 17.42%) as the top 3 recommendations provided by Buoy, followed by wait and watch (339/2141, 15.83%). Only 5.74% (123/2141) and 4.67% (100/2141) of users self-reported phone calls or in-person visits in the next 3 days and in-person visits that day or as soon as possible, respectively. The recommendations reported by Buoy closely matched primary care physicians in 2 weeks (924/2437, 37.91%), self-treatment (552/2437, 22.65%), and primary care in 1 to 2 days (456/2437, 18.71%). Most users (2098/2437, 86.09%) had 2 recommendations. Approximately 71.85% (1751/2437) had 3 recommendations, as reported by Buoy.

### Users’ Perceptions of the Web-Based Symptom Checking Experience

Users generally reported high levels of confidence in Buoy (mean 3.47, SD 0.97), found it useful (mean 4.18, SD 0.81) and easy to understand (mean 4.64, SD 0.53), and said that Buoy made them feel less anxious (mean 3.60, SD 1.05) and more empowered to seek medical help (mean 3.75, SD 0.96) Compared with White users, Latino and Black users had significantly more confidence in Buoy (*P*<.05), and the former also found it significantly more useful (*P*<.05; Table 3). Consistent with prior studies on trust in web-based health information sources [46-48], physicians were the most trusted source. However, Buoy was trusted more (mean 3.68, SD 0.61) than any other nonmedical source, including government agencies (mean 2.85, SD 0.95), family (mean 2.64, SD 0.76), and Google (mean 2.52, SD 0.79).

**Table 3:** Buoy user experience and recommendations (N=2437).

Item	Overall	Male (n=368)	Female (n=2069)	White (n=1227)	Latino (n=139)	Black (n=189)	Asian or Pacific Islander (n=86)
Comprehensibility of Buoy, mean (SD)	4.64 (0.53)	4.61 (0.49)	4.65 (0.53)	4.67 (0.50)	4.68 (0.55)	4.67 (0.53)	4.60 (0.45)
Buoy website was understandable, mean (SD)	4.60 (0.61)	4.57 (0.54)	4.60 (0.62)	4.63 (0.57)	4.63 (0.67)	4.60 (0.63)	4.57 (0.50)
Buoy website was easy to read, mean (SD)	4.66 (0.56)	4.62 (0.52)	4.67 (0.57)^a^	4.68 (0.54)	4.69 (0.59)	4.71 (0.55)	4.64 (0.48)
Language used on the Buoy website was easy to understand, mean (SD)	4.68 (0.55)	4.65 (0.51)	4.68 (0.55)	4.70 (0.51)	4.71 (0.58)	4.70 (0.54)	4.59 (0.49)
Confidence in Buoy, mean (SD)	3.47 (0.96)	3.39 (0.89)	3.49 (0.99)	3.44 (0.96)^b^	3.69 (0.92)^b^	3.63 (1.04)	3.48 (0.88)
Confidence in diagnoses, mean (SD)	3.34 (1.05)	3.27 (0.97)	3.36 (1.06)	3.29 (1.05)^b,c^	3.58 (0.99)^b^	3.53 (1.11)^c^	3.35 (0.96)
Confidence in the recommendation, mean (SD)	3.60 (1.02)	3.52 (0.95)	3.62 (1.03)	3.60 (1.01)	3.79 (0.98)	3.73 (1.09)	3.60 (0.91)
Perceived utility of Buoy, mean (SD)	4.18 (0.81)	4.14 (0.77)	4.19 (0.82)	4.16 (0.80)^d^	4.43 (0.73)^d^	4.25 (0.86)	4.20 (0.76)
Buoy enabled me to diagnose my symptoms more quickly, mean (SD)	4.15 (0.85)	4.11 (0.81)	4.16 (0.86)	4.12 (0.84)^e^	4.45 (0.75)^e,f^	4.20 (0.92)^f^	4.19 (0.80)
Using Buoy made the diagnosis of my symptoms easier, mean (SD)	4.16 (0.86)	4.12 (0.81)	4.16 (0.87)	4.13 (0.85)^b^	4.38 (0.79)^b^	4.23 (0.91)	4.14 (0.81)
Overall, I found Buoy useful to diagnose my symptoms, mean (SD)	4.23 (0.86)	4.19 (0.83)	4.24 (0.86)	4.22 (0.85)^b^	4.47 (0.75)^b^	4.31 (0.89)	4.27 (0.77)
Emotional consequences of using Buoy, mean (SD)	3.68 (0.90)	3.65 (0.79)	3.68 (0.91)	3.65 (0.88)	3.76 (1.02)	3.72 (1.00)	3.76 (0.66)
Less anxious, mean (SD)	3.60 (1.05)	3.56 (0.94)	3.61 (1.07)	3.58 (1.04)	3.70 (1.15)	3.59 (1.16)	3.67 (0.79)
Encouraged to seek help, mean (SD)	3.75 (0.96)	3.74 (0.88)	3.76 (0.98)	3.73 (0.95)	3.83 (1.11)	3.86 (1.05)	3.84 (0.76)

^a^Significant difference between sex (*P*<.05).

^b^Significant difference between White and Latino users (*P*<.05).

^c^Significant difference between White and Black users (*P*<.05).

^d^Significant difference between White and Latino users (*P*<.001).

^e^Significant difference between White and Latino users (*P*<.001).

^f^Significant difference between Latino and Black users (*P*<.05).

### Relationship Between Perceptions of a Web-Based Symptom Checker and Intention to Follow Recommended Actions

Overall, most users reported intentions to follow Buoy’s recommendations (1428/1886, 75.71%) and discuss Buoy’s recommendations with a physician or health care professional (1198/1830, 65.44%; Table 4). Users reported the strongest intention to follow Buoy’s wait and watch recommendation (mean 4.38, SD 0.90), followed by self-treatment (mean 4.33, SD 0.93), in-person visit that day or as soon as possible (mean 4.17, SD 1.01), phone call or in-person visit in the next 3 days (mean 4.05, SD 1.05), primary care physician in 2 weeks (mean 3.92, SD 1.19), and primary care physician in 1 to 2 days (mean 3.68, SD 1.26).

Intention to discuss Buoy’s recommendations was positively associated with having a regular provider (odds ratio [OR] 1.37, 95% CI 1.04-1.82), and an income >US $50,000 was negatively associated (OR 0.75, 95% CI 0.57-0.98; OR 66, 95% CI 0.48-0.91; Table 5). Users aged between 35 and 44 years (OR 1.51, 95% CI 1.13-2.03) and 45 and 64 years (OR 1.57, 95% CI 1.18-2.10) had better intentions of discussing recommendations than younger users (aged 18-34 years). Compared with White users, Latino (OR 1.96, 95% CI 1.22-3.25) and Black (OR 2.37, 95% CI 1.57-3.66) users had stronger intentions to discuss recommendations with a provider, and Black users were twice as likely to intend to do so. Confidence in Buoy (OR 1.54, 95% CI 1.34-1.76), perceived utility (OR 1.32, 95% CI 1.10-1.58), and anxiety reduction because of using Buoy (OR 1.43, 95% CI 1.24-1.63) were associated with higher intention to discuss Buoy’s recommendations.

Overall, users had strong intentions to follow Buoy’s recommendations, and users who self-reported very good or excellent health had the strongest intention to wait or watch or self-treat (OR 1.92, 95% CI 1.04-3.65; Table 5). Those who reported Buoy as easy to read and understand were 2.2 times (95% CI 1.21-4.14) more likely to intend to wait or watch or self-treat than those who reported lower comprehensibility for Buoy. Users with health insurance (OR 2.21, 95% CI 1.36-3.57) and a regular provider (OR 1.59, 95% CI 1.11-2.28) had the strongest intentions to seek care. Confidence in Buoy (OR 1.87, 95% CI 1.56-2.25) and anxiety reduction because of Buoy (OR 1.54, 95% CI 1.29-1.83) were also associated with a higher intention to seek care.

**Table 4:** Intentions to follow and discuss Buoy recommendations (N=2437).

Item		Overall	Male (n=368)	Female (n=2069)	White (n=1227)	Latino (n=139)	Black (n=189)	Asian or Pacific Islander (n=86)
**Intentions to follow Buoy’s recommendations (n=1886), n (%)**		1428 (75.71)	187 (9.91)	1241 (65.8)	908 (48.14)	116 (6.15)	149 (7.9)	62 (3.29)
	Wait and watch (n=283), n (%)	249 (87.9)	24 (9.6)	225 (79.5)	146 (51.6)	23 (8.1)	29 (10.2)	22 (7.8)
	Self-treatment (n=385), n (%)	339 (88.1)	50 (14.7)	289 (75.1)	226 (58.7)	34 (8.8)	32 (8.3)	8 (2.1)
	Phone call or in-person visit in the next 3 days (n=107), n (%)	81 (75.7)	14 (13.1)	67 (62.6)	49 (45.8)	7 (6.5)	8 (7.5)	3 (2.8)
	Primary care physician in 2 weeks (n=688), n (%)	487 (70.7)	60 (12.3)	427 (62.1)	317 (46.1)	35 (5.1)	48 (7.0)	17 (2.5)
	Primary care physician in 1 to 2 days (n=336), n (%)	205 (61.0)	29 (14.1)	176 (52.4)	137 (40.8)	9 (2.7)	22 (6.5)	10 (3.0)
	In-person visit that day or as soon as possible (n=87), n (%)	67 (77.0)	10 (11.5)	57 (65.5)	33 (37.9)	8 (9.2)	10 (11.5)	2 (2.3)
Intentions to discuss Buoy’s recommendations (n=1830), n (%)		1198 (65.46)	156 (8.52)	1042 (56.94)	758 (41.42)	109 (5.96)	150 (8.19)	51 (2.79)

**Table 5:** Intentions to follow Buoy’s recommendations.

Predictors	Discuss Buoy’s recommendations			No medical intention			Medical intention	
	OR^a^	*P* value	OR		*P* value	OR		*P* value
Intercept	0.02 (0.01-0.06)	<.001^b^	0.04 (0.00-0.75)		.03^b^	0.02 (0.00-0.11)		<.001^b^
Age 35 to 44 years	1. 51 (1.13-2.03)	.006^b^	0.66 (0.32-1.40)		.28	1.09 (0.74-1.60)		.67
Age 45 to 64 years	1.57 (1.18-2.10)	.002^b^	0.57 (0.26-1.26)		.16	1.07 (0.74-1.55)		.70
Age ≥65 years	1.31 (0.79-2.21)	.30	0.97 (0.28-4.08)		.96	1.12 (0.58-2.27)		.74
Female	0.86 (0.62-1.20)	.39	0.79 (0.31-1.80)		.59	1.00 (0.65-1.54)		.99
Black	2.37 (1.57-3.66)	<.001^b^	0.62 (0.27-1.57)		.23	1.49 (0.89-2.54)		.14
Latino	1.96 (1.22-3.25)	.007^b^	1.56 (0.48-7.12)		.50	1.38 (0.74-2.68)		.33
Asian or Pacific Islander	1.04 (0.62-1.74)	.99	0.79 (0.24-3.23)		.72	0.82 (0.43-1.64)		.57
Other ethnicities	1.56 (0.80-3.18)	.20	0.67 (0.18-3.39)		.58	0.94 (0.41-2.28)		.89
Have insurance	0.79 (0.52-1.18)	.25	0.74 (0.24-2.01)		.57	2.21 (1.36-3.57)		.001^b^
Have regular provider	1.37 (1.04-1.82)	.03	0.51 (0.21-1.14)		.12	1.59 (1.11-2.28)		.01^b^
General health status: very good or excellent	1.09 (0.86-1.38)	.50	1.92 (1.04-3.65)		.04^b^	0.95 (0.70-1.29)		.73
Some college	0.95 (0.68-1.38)	.77	1.03 (0.41-2.46)		.95	0.89 (0.57-1.38)		.61
College degree	0.73 (0.52-1.04)	.08	0.54 (0.21-1.29)		.18	0.69 (0.43-1.08)		.11
US $50,000-99,999	0.75 (0.57-0.98)	.03^b^	1.55 (0.76-3.18)		.22	1.20 (0.85-1.70)		.31
≥US $100,000	0.66 (0.48-0.91)	.01^b^	1.74 (0.76-4.17)		.20	0.92 (0.61-1.38)		.68
Comprehensibility of Buoy	1.19 (0.93-1.53)	.17	2.24 (1.21-4.14)		.01^b^	0.90 (0.65-1.22)		.49
Confidence in Buoy	1.54 (1.34-1.76)	<.001^b^	2.23 (1.61-3.14)		<.001^b^	1.87 (1.56-2.25)		<.001^b^
Perceived utility of Buoy	1.32 (1.10-1.58)	.002^b^	1.02 (0.63-1.62)		.93	1.12 (0.90-1.39)		.32
Emotional consequences of using Buoy	1.43 (1.24-1.63)	<.001^b^	1.02 (0.66-1.53)		.93	1.54 (1.29-1.83)		<.001^b^

^a^OR: odds ratio.

^b^Significant association.

## Discussion

### Principal Findings

This study sought to understand who uses web-based AI-powered symptom checkers and for what purposes. The demographic profile of Buoy users was similar to that described in other studies of web-based health information seekers, suggesting that older, marginalized groups continue to be digitally excluded. Consistent with data on internet-based health-seeking behaviors more generally [49], most Buoy users were middle-aged (or younger), female, and highly educated. More research is needed to better understand older adults’ web-based health information–seeking behaviors and support their medical and health decisions [50]. Although a scoping review of articles examining AI-driven symptom checkers from various perspectives found that those who do not have access to health care services are more likely to use symptom checkers [51], Buoy users overwhelmingly reported having health insurance. This finding does not negate the possibility that users were motivated by financial considerations, as most contemporary health plans require an out-of-pocket copayment. Nevertheless, this suggests that other considerations such as convenience were also salient.

Along these lines, prior research has identified an association between stigmatizing conditions and the use of symptom checkers [51]. In this study, gynecological problems were among the top 3 symptom groups. Furthermore, across presenting symptoms or diagnoses, approximately 11.04% (269/2437) of the respondents were “too embarrassed” to seek in-person care. Taken together, these findings suggest that symptom checkers might be particularly useful for users affected by conditions considered personal, embarrassing, stigmatizing, not warranting the physician’s attention, or requiring potentially uncomfortable or psychologically stressful physical examinations (such as pelvic examinations).

In examining the reasons for using the tool, approximately one-third of the respondents had persistent symptoms that failed to resolve spontaneously, another one-third had new symptoms, and the rest either thought they did not need professional attention or (as mentioned previously) were too embarrassed to seek care. Thus, some patients used the symptom checker because they had significant health-related concerns; some because they lacked sufficient concern to warrant in-person care; and some because they had issues with perceived quality, cost, or convenience of available care or simply wanted a second opinion. Symptoms that persist longer than expected have been identified as strong drivers of health-related anxiety and, thus, health care use [52]. At the same time, valuing convenience and lack of trust in the health care system (factors that may be particularly prominent among young people and racially and ethnically minoritized groups, respectively) have been associated with a lower propensity to use formal health care services [53].

Regarding the user experience, users had high levels of confidence in Buoy and found it useful. Moreover, users trusted Buoy more than any other nonmedical source. Perceived confidence, utility, and trust were associated with a stronger intention to discuss Buoy’s recommendations with a physician. This finding is in line with a study examining patient perspectives on the usefulness of a symptom checker [11]. Most Buoy users found the tool useful for diagnosis, and most reported that they would use it again. Although the experiences of users who discussed recommendations with their physicians varied, most felt that physicians were open to discussing the results of the tool. This is an important finding, as users may not follow recommendations to seek care if they believe that acting on the advice of a symptom checker will be questioned or even belittled by their physician, regardless of their confidence in the tool.

This study ultimately advances the understanding of web-based health information–seeking behaviors and outcomes by linking objective measures of information seeking from a web-based AI-powered system with clinical and psychosocial outcomes. The results demonstrate the potential utility of an artificially powered web-based health information tool to improve outcomes for users. Symptom checkers have been described as a means of addressing the lack of access to physicians and reducing unnecessary office visits [4].

There is a lack of research on whether the use of symptom checkers translates into medical care–seeking behaviors [4,33]. Future research should examine the effects of such tools on medical care seeking, specifically how users interpret recommendations, whether the recommendations are followed, and how user responses vary among sociodemographic groups. For example, one might surmise that individuals with limited access to care or with prior negative health care experiences might be more likely to attend to, appreciate, and follow such recommendations than their more privileged counterparts. Although symptom checkers may empower users to make more informed decisions, they might paradoxically worsen health disparities if their use were less accessible to some groups. Currently, web-based symptom checkers are mostly available for free. As web-based symptom checker companies establish partnerships with employers and health insurance companies to ensure profits, not all users may be equally ready or able to pay for symptom checking.

### Limitations and Strengths

We partnered with the Buoy technical and medical staff to sample the users. Owing to our partnership approach, we were able to obtain the specific symptoms reported by the participants as the primary reason for using Buoy, as well as the possible diagnoses identified by Buoy and Buoy’s triage recommendation. This allowed for the comparison and validation of self-reported data. We also obtained from Buoy the symptoms, diagnoses, triage, and sex of eligible users who opted not to participate in our study. This allowed us to compare our sample to the population of users and assess potential bias. In addition, a benefit of a collaborative approach is the potential to overcome the self-report limitations of prior studies. Thus, we obtained from Buoy the paths that individuals took and Buoy’s final recommendation. We were also able to match the initial reason for the consultation to the reason reported in the survey and assess the extent to which respondents understood the recommendation and intended to act upon it. By leveraging a public or private partnership, we were able to explore the use and effects of a web-based symptom checker, which has important implications for health equity and the health care system during and after the COVID-19 crisis.

The limitations of this study include the use of cross-sectional data, which limited the ability to make any causal inferences, and the potential lack of applicability to other web-based symptom checkers. In addition, we did not assess the actual search terms entered by users. Finally, our study used a limited definition of web-based health information. Searches for symptoms using a web-based symptom checker differ from other forms of health-related information communicated through the internet. For example, web-based health communities can also be a source of social support [54] and peer-to-peer medical advice [7].

### Conclusions

The results of this study demonstrate the potential utility of a web-based health information tool to empower people to seek appropriate care and reduce health-related anxiety. An interactive symptom checker might provide more personalized and potentially reliable medical information than other forms of web-based health information–seeking. Despite encouraging results suggesting that the web-based tool may fulfill unmet health information needs among women and Black and Latino adults, analyses of the user base illustrate persistent second-level digital divide effects.

For web-based symptom checkers to make a meaningful contribution, they must not only be trusted by users but also meet their diverse needs, especially those concerning usability and comprehensibility. The inability to access web-based symptom checkers may also be associated with increased disparities in access to care, particularly among groups that have lagged historically in terms of digital access and literacy. Moreover, web-based symptom checker business models may further exacerbate these disparities. In contrast, AI technologies such as Buoy have the potential to alleviate disparities by allowing users to access accurate, actionable, and personalized advice within an evolving but often confusing web-based health information environment. Finally, there is a lack of evidence on whether web-based symptom checkers influence care-seeking behaviors. To address this gap, future research will use Buoy users’ follow-up data to assess the extent to which users discuss their web-based findings with physicians, as well as barriers to the same and patient satisfaction.

## Appendix

Multimedia Appendix 1 Buoy triage levels and recommendations.

Multimedia Appendix 2 Symptom and diagnosis codes and frequencies.

## Abbreviations

AI: artificial intelligence
NAMCS: National Ambulatory Medical Care Survey
OR: odds ratio
RQ: research question
