# Supplementary material for: Health Information Seeking From an Intelligent Web-Based Symptom Checker: Cross-sectional Questionnaire Study
Source: J Med Internet Res. 2022 Aug 19;24(8):e36322. doi: 10.2196/36322 (PMC9440406; doi:10.2196/36322)
Supplement: Multimedia Appendix 1 [file jmir_v24i8e36322_app1.doc]

**Appendix 1. Buoy triage levels and recommendations.**

| Triage Level | Display Name | Display Text | Explanation |
| --- | --- | --- | --- |
| Triage 1 | Wait and watch | “This condition usually resolves on its own. Over-the-counter products and at-home remedies can help you manage your symptoms.” | Includes matches that can be physiologic variations or matches that are not necessarily any expression of any pathology. Examples: benign skin lesions like lipomas. |
| Triage 2 | Self-treatment | “This condition usually resolves on its own. Over-the-counter products and at-home remedies can help you manage your symptoms.” | Includes condition matches that, with appropriate information, can be managed without involvement of a healthcare provider. These are usually conditions that mainly need symptomatic self-treatment. |
| Triage 3 | Phone call or in-person visit sometime in the next 3 days | “This condition is unlikely to escalate, but it should be evaluated for treatment within a few days. Options include telemedicine, a walk-in clinic, or calling your primary care doctor.” | Includes less urgent or serious matches, the conditions are unlikely to escalate quickly. Type of provider and existence of pre-existing relationship with the provider is less important. These condition matches can be seen in settings where specific resources (like X-ray) are not needed. Examples: ankle strain (for physical exam, Ottowa ankle rules), ear wax blockage, minor skin rashes or infections. |
| Triage 4 | See Primary care doctor within two weeks | “This condition is not urgent, but it requires attention. Your primary care doctor can evaluate, diagnose, and treat this condition, or they can help you coordinate further care if needed.” | Includes condition matches that probably need some more longer-term evaluation, guidance, and treatment. Not super urgent, no harm in waiting a little bit. Examples: skin changes that need biopsy, arthritis, some chronic diseases unlikely to escalate over the course of days/weeks. |
| Triage 5 | See primary care doctor within 1-2 days | “This condition should be looked at in the next day or two. Your primary care doctor can evaluate, diagnose, and treat this condition, or they can help you coordinate further care if needed.” | Includes conditions that need to be seen in a more timely manner to prevent escalation, and where there is a preference for evaluation by the primary care provider. Reason for this preference is the need for potential follow up by this provider or the need for more context (medical history) that can be derived from a pre-existing patient-provider relationship.  Examples: infectious diseases, potential malignancies, cellulitis. |
| Triage 6 | In-person visit today, or as soon as possible | “This condition is not life threatening, but it should be treated today. Options include visiting your primary care doctor, a walk-in clinic, or an urgent care center. It is not necessary to visit an ER.” | Includes conditions that need to be seen in a more timely manner to prevent escalation, without a specific preference for evaluation by the primary care provider. These would be conditions that are appropriate for urgent care clinics (or same day PCP evaluation), where there are more diagnostic and treatment resources available.  These conditions are usually more urgent than the conditions that are matched with triage category 4, but don't require the cost or sophistication of the ER. Examples: bacterial pneumonia, cuts and wounds, influenza in vulnerable populations. |
| Triage 7^a^ | Hospital emergency room | “This condition requires immediate medical care.” | Includes condition matches that need the sophistication of an emergency room and are associated with potential hospital admission. Examples: fractures, more serious infections, appendicitis, cholecystitis, deep venous thrombosis. |
| Triage 8^a^ | Emergency medical service | “This condition is an emergency that could be life threatening. Consider calling 911.” | Explanation: includes condition matches that require immediate care, and where minutes to hours make the difference. Examples: heart attack, intracerebral hemorrhage (brain bleed), stroke, severe shortness of breath. |

^a^For ethical reasons, users who were advised by Buoy to seek immediate medical care, including immediate medical care via 911 or in the emergency department were excluded from eligibility.
