# Supplementary material for: Health Information Seeking From an Intelligent Web-Based Symptom Checker: Cross-sectional Questionnaire Study
Source: J Med Internet Res. 2022 Aug 19;24(8):e36322. doi: 10.2196/36322 (PMC9440406; doi:10.2196/36322)
Supplement: Multimedia Appendix 2 [file jmir_v24i8e36322_app2.docx]

**Appendix 2. Symptom and diagnosis codes and frequencies.^a^**

|  |  |  | **Analytic**  **Sample**^a^ |  |  |  | **Eligible**  **Opt-Outs** |
| --- | --- | --- | --- | --- | --- | --- | --- |
| N of participants |  |  | *N*=2,437 |  |  |  | *N*=27,816 |
|  |  |  | *Proportion (95% CI)* |  |  |  | *Proportion (95% CI)* |
|  |  |  |  |  |  |  |  |
| *N* of unique symptoms |  |  | 731 |  |  |  | 1561 |
| First Symptom^b^ | N |  |  |  | N |  |  |
| General Symptoms | 318 |  | 0.13 (0.12, 0.14) |  | 4194 |  | 0.15 (0.15, 0.15) |
| Psychological and Mental Health | 44 |  | 0.02 (0.01, 0.02) |  | 410 |  | 0.01 (0.01, 0.02) |
| Nervous System | 194 |  | 0.08 (0.07, 0.09) |  | 1930 |  | 0.07 (0.07, 0.07) |
| Cardiovascular and Lymphatic Systems | 3 |  | 0 (0, 0) |  | 100 |  | 0 (0, 0) |
| Eyes and Ears | 102 |  | 0.04 (0.03, 0.05) |  | 959 |  | 0.03 (0.03, 0.04) |
| Respiratory System | 172 |  | 0.07 (0.06, 0.08) |  | 1729 |  | 0.06 (0.06, 0.06) |
| Digestive System | 333 |  | 0.14 (0.12, 0.15) |  | 3434 |  | 0.12 (0.12, 0.13) |
| Urinary System | 92 |  | 0.04 (0.03, 0.05) |  | 1011 |  | 0.04 (0.03, 0.04) |
| Male Reproductive System | 8 |  | 0 (0, 0.01) |  | 329 |  | 0.01 (0.01, 0.01) |
| Female Reproductive System | 366 |  | 0.15 (0.14, 0.16) |  | 4897 |  | 0.18 (0.17, 0.18) |
| Skin, Nails and Hair | 185 |  | 0.08 (0.07, 0.09) |  | 2336 |  | 0.08 (0.08, 0.09) |
| Musculoskeletal System | 591 |  | 0.24 (0.23, 0.26) |  | 6196 |  | 0.22 (0.22, 0.23) |
| Injury And Adverse Effects | 4 |  | 0 (0, 0) |  | 87 |  | 0 (0, 0) |
| Uncodable | 25 |  | 0.01 (0.01, 0.01) |  | 204 |  | 0.01 (0.01, 0.01) |
|  |  |  |  |  |  |  |  |
| *N* of participants with serious first symptom | 118 |  |  |  | 1344 |  |  |
| Serious First Symptom^b^ |  |  |  |  |  |  |  |
| Chest Pain, Heart Related | 58 |  | 0.49 (0.4, 0.58) |  | 767 |  | 0.57 (0.54, 0.6) |
| Bleeding | 7 |  | 0.06 (0.02, 0.1) |  | 92 |  | 0.07 (0.05, 0.08) |
| Loss Of Consciousness | 0 |  | 0 (0, 0) |  | 0 |  | 0 (0, 0) |
| Shortness Of Breath | 15 |  | 0.13 (0.07, 0.19) |  | 161 |  | 0.12 (0.1, 0.14) |
| Weight Loss | 0 |  | 0 (0, 0) |  | 0 |  | 0 (0, 0) |
| Other | 38 |  | 0.32 (0.24, 0.41) |  | 324 |  | 0.24 (0.22, 0.26) |
|  |  |  |  |  |  |  |  |
| *N* of unique diagnoses |  |  | 370 |  |  |  | 980 |
| First Diagnosis Display Name^c^ |  |  |  |  |  |  |  |
| Infective and Parasitic Diseases | 255 |  | 0.1 (0.09, 0.12) |  | 1960 |  | 0.07 (0.07, 0.07) |
| Neoplasms | 125 |  | 0.05 (0.04, 0.06) |  | 419 |  | 0.02 (0.01, 0.02) |
| Endocrine, Nutritional, and Metabolic Diseases | 189 |  | 0.08 (0.07, 0.09) |  | 2490 |  | 0.09 (0.09, 0.09) |
| Diseases of the Blood and Blood-Forming Organs | 22 |  | 0.01 (0.01, 0.01) |  | 285 |  | 0.01 (0.01, 0.01) |
| Mental Disorders | 76 |  | 0.03 (0.02, 0.04) |  | 985 |  | 0.04 (0.03, 0.04) |
| Diseases of the Nervous System | 164 |  | 0.07 (0.06, 0.08) |  | 1547 |  | 0.06 (0.05, 0.06) |
| Diseases of the Eye | 19 |  | 0.01 (0, 0.01) |  | 192 |  | 0.01 (0.01, 0.01) |
| Diseases of the Ear | 32 |  | 0.01 (0.01, 0.02) |  | 213 |  | 0.01 (0.01, 0.01) |
| Diseases of the Circulatory System | 70 |  | 0.03 (0.02, 0.04) |  | 729 |  | 0.03 (0.02, 0.03) |
| Diseases of the Respiratory System | 87 |  | 0.04 (0.03, 0.04) |  | 1678 |  | 0.06 (0.06, 0.06) |
| Diseases of the Digestive System | 222 |  | 0.09 (0.08, 0.1) |  | 2556 |  | 0.09 (0.09, 0.1) |
| Diseases of the Urinary System | 82 |  | 0.03 (0.03, 0.04) |  | 885 |  | 0.03 (0.03, 0.03) |
| Male Reproductive System | 12 |  | 0 (0, 0.01) |  | 244 |  | 0.01 (0.01, 0.01) |
| Female Reproductive System | 244 |  | 0.1 (0.09, 0.11) |  | 2963 |  | 0.11 (0.1, 0.11) |
| Diseases of the Skin and Subcutaneous Tissue | 321 |  | 0.13 (0.12, 0.15) |  | 4250 |  | 0.15 (0.15, 0.16) |
| Diseases of the Musculoskeletal System and Connective Tissue | 366 |  | 0.15 (0.14, 0.16) |  | 5038 |  | 0.18 (0.18, 0.19) |
| Congenital Anomalies | 6 |  |  |  | 18 |  |  |
| Perinatal Morbidity and Mortality Conditions | 0 |  | 0 (0, 0) |  | 0 |  | 0 (0, 0) |
| Injuries and adverse effects | 118 |  | 0.05 (0.04, 0.06) |  | 976 |  | 0.04 (0.03, 0.04) |
| General Symptoms | 27 |  | 0.01 (0.01, 0.02) |  | 387 |  | 0.01 (0.01, 0.02) |
| Uncodable | 0 |  |  |  | 1 |  |  |

^a^The number of Buoy users in the analytic sample was 2,437; during the period of the study, there were a total of 27,816 potentially eligible users (aged 18 and older, US IP address, those seeking for themselves, and who completed the Buoy interview in <10 minutes) who opted not to participate. Users entered up to five presenting symptoms; Buoy Health provided all five as reflected in the Buoy database of medical concepts. For this analysis, we report only the first, because that was the primary issue driving use of the online symptom checker. We coded the symptoms using the CDC’s National Center for Health Statistics’ National Ambulatory Medical Care Survey (NAMCS) Codebook.[42]

^b^Using the NAMCS[42] codebook, we adapted Shapiro and colleagues’ [51] categorization of whether a particular symptom was serious and likely to require medical attention.

^c^At the completion of each interview, Buoy provides users with up to three possible diagnoses – ranked and weighted by probability and stability of probability estimates, according to their propriety algorithm – along with recommendations for next actions. We coded the diagnoses into major systems, disorders, and conditions according to NAMCS.
